# Overweight dogs exercise less frequently and for shorter periods: results of a large online survey of dog owners from the UK

**DOI:** 10.1017/jns.2017.6

**Published:** 2017-04-17

**Authors:** Alexander J. German, Emily Blackwell, Mark Evans, Carri Westgarth

**Affiliations:** 1 Institute of Ageing and Chronic Disease, University of Liverpool, Neston, UK; 2 School of Clinical Veterinary Science, University of Bristol, Langford, UK; 3 Independent Veterinary Consultant, Guildford, UK; 4 Institute of Infection and Global Health, University of Liverpool, Liverpool, UK

**Keywords:** Obese dogs, Canine nutrition, Physical activity, Dog walking

## Abstract

Canine obesity is now the number one health concern in dogs worldwide. Regular physical activity can improve health, and owners are advised to exercise their dogs on a regular basis. However, limited information exists about associations between overweight status of dogs and walking activity. An online survey was conducted between June and August in 2014, coinciding with the broadcast of a national UK television programme, exploring dog behaviour. Information gathered included signalment, overweight status, and owner-reported information on duration and frequency of dog walking. The University of Liverpool Ethics Committee approved the project, and owners consented to data use. Simple and multiple logistic regression analyses were used to determine associations between overweight status and dog walking activity. Data were available from 11 154 adult dogs, and 1801 (16·1 %) of these were reported as overweight by their owners. Dogs reported to be overweight dogs were more likely to be neutered (*P* < 0·0001) and older (*P* < 0·0001). Various breeds were over-represented including beagle, Cavalier King Charles spaniel, golden retriever, Labrador retriever and pug (*P* < 0·0001 for all). Both frequency and duration of walking were negatively associated with overweight status (*P* < 0·0001 for both). On multiple regression analysis, duration and frequency were independently and negatively associated with the odds of being overweight, along with a range of other factors including age, neuter status and breed. This study has identified associations between overweight status and exercise. In the future, studies should determine the reason for this association, and whether changes in walking activity can influence weight status.

Overweight and obesity are common in dogs^(^^1^^,^^2^^)^, and recent evidence suggests that the prevalence is steadily increasing^(^^3^^)^. Known co-morbidities include osteoarthritis, diabetes mellitus, respiratory disease and neoplasia^(^^1^^,^^4^^)^. Dogs that are overweight also develop metabolic derangements^(^^5^^)^, and altered respiratory function^(^^6^^)^. Studies have also demonstrated that obese dogs have a poorer quality of life^(^^7^^)^, and that dogs that are overfed and are overweight long term tend to have a shorter lifespan^(^^8^^)^. Numerous factors are known to predispose to obesity including breed, sex and being neutered^(^^1^^)^. Factors relating to the owner and environment are also known to be important, including their income, being middle aged, living in a single-dog household and feeding snacks^(^^2^^,^^9^^)^.

Physical activity is also a possible risk factor for weight gain, not least given that research suggests that obesity decreases physical activity in humans^(^^10^^,^^11^^)^. Conversely, other studies have suggested that low physical activity might predispose to weight gain, which then reduces physical activity^(^^12^^)^. To date, few studies have examined associations between activity and obesity in dogs. In two previous owner surveys of risk factors associated with obesity, the risk of being overweight declined steadily for each 1 h of exercise per week undertaken^(^^2^^,^^9^^)^. However, the relationship between duration and frequency was not considered. The aim of the present study was to examine patterns of exercise and their relationship with overweight status in dogs from a large online survey of owners.

## Methods and materials

### Study design and methodology

A television documentary series called ‘Dogs – Their Secret Lives’ was produced during early 2014, and aired on Channel 4 television in the same year. It covered aspects of health and wellbeing in the UK canine population. The series featured three of the study authors (A. J. G., E. B., M. E.), and was broadcast during the summer months. To accompany the series, an online survey was conducted between June and August in 2014, and this coincided with when the episodes aired. The University of Liverpool Ethics Committee approved the study, and all owner participation was voluntary, whereby owners who wished to complete the survey logged onto the Channel 4 website. Further, owners gave permission for their data to be used, in a fully anonymised form (i.e. any client-identifying data removed), and for the results to be publicised both on the television shows and online. Further, they were not required to answer questions that they were unclear about, or did not wish to answer. To be eligible for inclusion in the data analysis part of the study, dogs had to be adult (≥2 years of age) and questionnaire information needed to be complete, i.e. all questions used in the present study needed to be answered.

### Survey design

There were forty-three questions in the survey, with four covering personal data not used in the project. The remaining questions covered signalment details of the dog (age, sex, neuter status, breed), current body weight, whether or not the dog was overweight, lifestyle, activity and behaviour. Owners gave their responses in free text boxes for age and body weight, and either checked boxes or used drop-down menus for the remaining questions. For overweight status, owners responded to the question, ‘Is your dog overweight?’, with their answer being based on their own subjective impression (i.e. no reference to a formal body condition scoring system). The main questions considered in the present study were those relating to activity, whereby two questions were considered. For exercise frequency, the question asked was ‘How often do you exercise your dog outside of your home or garden?’ and respondents could select: ‘more than once a day’, ‘once a day’, ‘4–6 times per week’, ‘1–3 times per week’ or ‘never’. For exercise duration, the question asked was ‘Each time you exercise your dog how long is it for?’ and respondents could select: ‘over an hour’, ‘30 minutes to an hour’, ‘11–30 minutes’ and ‘0–10 minutes’. The same data on exercise were also used for a separate study examining activity patterns amongst different dog breeds^(^^13^^)^, whilst the questions relating to behaviour are reported elsewhere^(^^14^^)^.

### Data handling and statistical analysis

All data were first entered into a computer spreadsheet (Excel version 14; Microsoft), to enable data checking and cleaning. Details of the data-cleaning process are reported elsewhere^(^^14^^)^. Briefly, data were removed from all dogs under 2 years of age (to ensure no growing dogs were included), dogs with any missing data and dogs with obvious errors in the dataset. Subsequently, age and body-weight data inaccuracies were checked more closely given that these were free text boxes and more liable to errors. This involved using the ‘sort’ function to check age and weight data within breed and sex, with expected ranges for the respective breed based upon information reported in an online encyclopaedia (https://www.wikipedia.org). Dogs with ages more than 20 % outside the range reported for each breed (given uncertainties of the reported age ranges) were removed. Computer software (Stats Direct version 3.0.171; Stats Direct Ltd) was used for all tests. Statistical significance was considered when *P* < 0·05, for two-sided analyses.

Initially, associations between overweight status and activity levels were examined using *χ*^2^ tests for trend; frequency and duration of exercise were examined separately, and the test was applied across the ordered categories (e.g. from least frequent exercise category to most frequent exercise category and from shortest duration of exercise to longest duration of exercise). Associations were further explored using logistic regression analysis to determine differences amongst exercise categories, and the possible influence of confounding variables (e.g. signalment). The outcome variable was overweight status, whilst variables considered were activity frequency, activity duration and signalment (e.g. age, sex, neuter status and breed). For activity frequency, ‘more than once a day’ was used as the reference category and, for activity duration, ‘over an hour’ was used as the reference category. Sex and neuter status were binary and age continuous as whole years. For breed, those that had previously been identified as significantly associated with overweight status (at *P* < 0·0017) in a study using the same data^(^^14^^)^ were initially included, each coded as a separate variable (whereby 1 = from that breed and 0 = not from that breed). An initial multiple regression model was constructed including all variables, and this was refined in a forwards and backwards stepwise fashion, so as to optimise the fit of the model and take account of covariance. During this procedure, exercise frequency and activity were retained *en bloc* even when single categories were not significant. When decisions were made regarding which breeds to retain and discard, priority was given to those breeds with the largest numbers. In the final model, only variables that were significant in their own right (at *P* < 0·05) were retained.

## Results

### Final dataset

Details of the demographics of the final dataset are reported elsewhere^(^^14^^)^. Briefly, there were 17 028 survey responses available and, after the data-cleaning steps, responses for 11 154 dogs were used in the final analysis. A range of breeds was represented, with the most common being Labrador retriever (1344), Jack Russell terrier (606), Border collie (583) and cocker spaniel (512). The median age of the population was 5 (range 2–19) years and median body weight was 20 (range 1–107) kg. A total of 1801 owners (16·1 %) reported that their dog was overweight, and these dogs were significantly heavier, more likely to be neutered, and of certain breeds (e.g. beagle, bull terrier, bulldog, Cavalier King Charles spaniel, Chihuahua, golden retriever, Labrador retriever and pug) and older^(^^14^^)^.

### Association between overweight status and activity level

Owner-reported exercise frequency and duration are shown in Table 1. With simple logistic regression analysis, dogs that were reported to be overweight exercised less frequently (*P* < 0·0001) and for a shorter time (*P* < 0·0001) than those not reported to be overweight. On multiple logistic regression analysis, both exercise frequency and duration were independently and negatively associated with overweight status along with a range of other factors including age, neuter status and breed (Table 2). Compared with dogs that were exercised more than once per d, the odds of being overweight steadily increased for dogs that were exercised 4–6 times per week (OR 1·297; *P* = 0·0165), 1–3 times per week (OR 1·633; *P* < 0·0001) and not exercised at all (OR 1·975; *P* = 0·0087). However, there was no difference between dogs exercised daily and more frequently (*P* = 0·1270). Further, compared with dogs exercised for over 1 h, the odds of being overweight increased steadily for dogs exercised for 30 min to 1 h (OR 1·266; *P* = 0·0031), 11–30 min (OR 1·754; *P* < 0·0001) and 0–10 min (OR 2·241; *P* < 0·0001).

**Table 1.** Exercise frequency and duration in the study dogs (Numbers and percentages)

	Owner-reported weight status				
	Overweight (*n* 1801)		Not overweight (*n* 9353)		
Exercise	*n*	%	*n*	%	P*
Frequency					<0·0001
More than once per d	948	52·6	5347	57·2	
Once per d	538	29·9	2823	30·2	
4–6 times per week	124	6·9	556	5·9	
1–3 times per week	163	9·1	565	6·0	
Never	28	1·6	62	0·7	
Duration					<0·0001
Over 1 h	236	13·1	1735	18·5	
30 min to 1 h	903	50·1	5084	54·4	
11–30 min	608	33·8	2395	25·6	
0–10 min	54	3·0	140	1·5	

* *χ*^2^ Test for linear trend applied across ordered categories.

**Table 2.** Multiple logistic regression analysis on associations between overweight status and both signalment factors and activity (Odds ratios and 95 % confidence intervals)

Variable	OR	95 % CI	*P*
Sex			
Female (reference)	1·000	–	–
Male	0·854	0·770, 0·948	0·0029
Neuter status			
Intact (reference)	1·000	–	–
Neutered	2·212	1·870, 3·426	<0·0001
Age (per year)	1·063	1·046, 1·079	<0·0001
Breed			
Beagle	8·100	5·485, 11·961	<0·0001
Cavalier King Charles spaniel	2·254	1·883, 3·426	<0·0001
Golden retriever	1·893	1·409, 2·542	<0·0001
Labrador retriever	1·736	1·500, 2·009	<0·0001
Pug	4·878	2·508, 9·369	<0·0001
Exercise frequency			
More than once per d (reference)	1·000	–	–
Once per d	1·097	0·974, 1·235	0·1270
4–6 times per week	1·297	1·049, 1·603	0·0165
1–3 times per week	1·633	1·344, 1·984	<0·0001
Never	1·975	1·188, 3·283	0·0087
Exercise duration			
Over 1 h (reference)	1·000	–	–
30 min to 1 h	1·266	1·083, 1·481	0·0031
11–30 min	1·754	1·482, 2·076	<0·0001
0–10 min	2·241	1·531, 3·281	<0·0001

## Discussion

The present study involved surveying the opinions of a large number of owners regarding patterns of exercise in their dogs. To our knowledge, it is the largest survey of its kind ever conducted that has explored associations between overweight status and activity. Independent associations were identified for both exercise frequency and exercise duration with overweight status, and these remained when possible confounding factors (such as breed, age, sex and neuter status) were taken into account. As a result, the findings of the study extend those of previous epidemiological surveys, which have only previously demonstrated an association with total weekly activity^(^^2^^,^^9^^)^. They also support the findings of a small study, which used objectively measured physical activity with accelerometers^(^^15^^)^. In that study, dogs that were obese had lower levels of vigorous intensity activity than dogs that were not obese. Of course, a limitation of both of these studies is that neither has investigated the reasons for the association. As a result, prospective studies are now required to investigate further the link between activity and overweight status.

Dogs that were reported to be overweight exercised less frequently, with the odds of being overweight steadily increasing when they were exercised less than once per d. Furthermore, duration of activity was negatively associated with being overweight, with the odds of being overweight steadily increasing the shorter the duration of exercise. As mentioned above, the given the nature of the study, it is not possible to determine the reason for the association, whether it is causal, and any direction to the causality. For example, it is possible that dogs that are overweight are less able to exercise than those that are not, with the result that they take less frequent and shorter periods of exercise. Alternatively, it is possible that dogs exercising less are more prone to weight gain, and becoming overweight. If this is the case, the results of the present study suggest that a suitable target for dog owners should be to exercise their dog at least every day and for as long as possible.

There are a number of limitations to consider with this study. First, the proportion of overweight dogs in the population examined was lower than expected (16·1 %) based upon the expected prevalence of overweight dogs in the UK^(^^2^^)^. The possible reasons for this association have been discussed before^(^^14^^)^, and most likely to be due either to participation bias, whereby the owners who took part were not representative of the UK population as a whole, or due to owners underestimating actual body condition, meaning that many overweight dogs were incorrectly classified as normal. The resulting impact of this is that magnitude of the associations identified may well have appeared weaker than they actually were. A second limitation is the fact that activity was assessed on the basis of owner reports, rather than more objective measures, for example using accelerometers. This may have led to inaccuracies in that they are based upon what owners think and say they do, rather than what they actually do. This may well have been compounded by the fact that response options were limited, and owners were required to choose from a defined number of categories. Some owners might have found it difficult to select an appropriate category, for example, if their dog undertook a pattern of exercise that varied from day to day in terms of frequency and duration. A final limitation was the fact that, whilst a number of confounding variables were examined, others were not considered including owner factors (e.g. ability to take their dog for a walk), local environment, local weather patterns and concurrent illness (e.g. orthopaedic disease). Size of dog has previously been associated with motivation to take dogs for walks^(^^16^^)^, but this was not included in the model due to collinearity with breed. Further studies should consider acquiring more detail on owners and their dogs, further explore further different activity patterns, and consider assessing physical activity in different ways concurrently, perhaps by using owner reports in conjunction with objective measurements of physical activity. Ultimately, intervention studies will probably be required to determine reasons for the association between overweight status and both the duration and frequency of exercise.

## Conclusion

The findings of the present study have identified associations between dogs that are overweight and both their frequency and duration of exercise. The odds of being overweight were greater both for dogs that exercised less than once per d and those that exercised for less than 1 h each time. Future studies should determine the reason for this association, and whether changes in exercise pattern can influence the likelihood of dogs becoming overweight.
